# Sjögren’s Syndrome Antigen B Acts as an Endogenous Danger Molecule to Induce Interleukin-8 Gene Expression in Polymorphonuclear Neutrophils

**DOI:** 10.1371/journal.pone.0125501

**Published:** 2015-04-27

**Authors:** Cheng-Han Wu, Ko-Jen Li, Chia-Li Yu, Chang-Youh Tsai, Song-Chou Hsieh

**Affiliations:** 1 Graduate Institute of Clinical Medicine, National Taiwan University College of Medicine, Taipei, Taiwan; 2 Institute of Clinical Medicine, National Yang-Ming University, Taipei, Taiwan; 3 Division of Immunology, Rheumatology and Allergy, Department of Internal Medicine, National Taiwan University Hospital and National Taiwan University College of Medicine, Taipei, Taiwan; 4 Institute of Molecular Medicine, National Taiwan University College of Medicine, Taipei, Taiwan; 5 Section of Allergy, Immunology and Rheumatology, Taipei Veterans General Hospital, Taipei, Taiwan; Queen Mary University of London, UNITED KINGDOM

## Abstract

**Background:**

Sjögren’s syndrome antigen B is expressed in the nucleus and surface membrane of human polymorphonuclear neutrophils and is released after cell death. However, its biological role is not clear. This study is aimed to investigate the effect of Sjögren’s syndrome antigen B on human polymorphonuclear neutrophils.

**Methods:**

Human recombinant Sjögren’s syndrome antigen B (rSSB) purified from *E*. *coli* was incubated with human polymorphonuclear neutrophils as well as retinoid acid-induced granulocytic differentiated HL-60 cells, HL-60 (RA). Interleukin (IL)-8 protein production and mRNA expressions were measured by enzyme-linked immunosorbent assay and quantitative-polymerase chain reaction, respectively. Uptake of fluorescein isothiocyanate (FITC)-rSSB was assessed by flow cytometry and fluorescence microscopy. Moreover, mitogen-activated protein kinase (MAPK) pathways and nuclear factor-kappaB activation were investigated.

**Results:**

Human rSSB stimulated IL-8 production from normal human neutrophils and HL-60 (RA) cells in a time- and dose-dependent manner. This IL-8-stimulated activity was blocked by chloroquine and NH4Cl, indicating that endosomal acidification is important for this effect. We found rSSB activated both MAPK pathway and nuclear factor-kappaB signaling to transcribe the IL-8 gene expression of cells. Furthermore, tumor necrosis factor-α exerted an additive effect and rSSB-anti-SSB immune complex exhibited a synergistic effect on rSSB-induced IL-8 production.

**Conclusions:**

Sjögren’s syndrome antigen B might act as an endogenous danger molecule to enhance IL-8 gene expression in human polymorphonuclear neutrophils.

## Introduction

Autoimmune diseases originating from breakdown of self-tolerance are frequently associated with antigen-driven autoantibody production [1]. La or Sjögren’s syndrome antigen B (SSB), a ribonucleoprotein with a molecular weight around 48 kDa, has been reported to be involved in the maturation of RNA polymerase III transcripts [2,3], small RNA biogenesis, translation of specific viral and cellular mRNAs [3], and to be associated with human telomerase complex, thus influencing telomere homeostasis [4]. Autoantibodies against the La/SSB complex (anti-SSB) have frequently been reported in patients with systemic lupus erythematosus (SLE) and primary Sjögren’s syndrome (pSS) [5].

Polymorphonuclear neutrophils (PMNs), the most abundant blood leukocytes, are important innate immune cells that act as the first line of body defense. In response to infections or tissue injury, neutrophils migrate to the sites of involvement within minutes [6]. The infiltrated PMNs initiate inflammatory responses and produce a number of cytokines and chemokines including tumor necrosis factor (TNF)-α, interleukin (IL)-1, and the highly neutrophil-specific chemoattractant IL-8 [7,8]. These mediators further prime neutrophils and stimulate many effector functions such as adhesion and chemotaxis, respiratory burst, release of granule enzymes, inhibition of apoptosis, and autocrine/paracrine secretion of IL-8 [9,10]. These effects may amplify an inflammatory loop and accelerate tissue damage and cell death.

Neutrophils [11] and inflammation [12–15] have been involved in the development of autoimmunity in genetically susceptible subjects. Moreover, inflammation-induced excessive cell death plays an important role in the induction of autoimmunity [16–18]. Many intracellular autoantigens and endogenous adjuvants such as heat shock proteins, uric acid, ATP and UTP, are released during cell death [19–22]. These released endogenous adjuvants act as danger molecules to alert the immune system by recruiting and activating innate and adaptive immune cells to protect the body, but can concomitantly lead to the exposed autoantigens becoming immunogenic [19]. Furthermore, some autoantigens act as immunomodulators or chemoattractants to drive pathologic immune responses in systemic autoimmune diseases [23–29]. These observations suggest that endogenous autoantigens *per se* may play an active role in autoimmune pathogenesis.

Our previous study demonstrated that La/SSB was expressed in the nucleus and surface membrane of human PMNs and that purified anti-SSB antibody concomitantly increased apoptosis and IL-8 production of human PMNs [30]. However, effect of La/SSB on human PMNs is not clear. As La/SSB is released upon cell death, it would be interesting to know whether La/SSB *per se* acts as an endogenous adjuvant to modulate PMN functions. In this study, we found recombinant SSB (rSSB) activated both MAPK pathway and nuclear factor-kappaB (NF-κB) signaling to transcribe the IL-8 gene expression of human neutrophils. Moreover, this IL-8-stimulated activity was blocked by chloroquine and NH4Cl, indicating that endosomal acidification is important for this effect.

## Material and Methods

### Patients and controls

Patients with SLE [31] and normal individuals were included in this study. Human PMNs were isolated as described in our previous study [30]. This study was approved by the Research Ethics Committee of National Taiwan University Hospital, Taipei, Taiwan. Written informed consent was obtained from each participant.

### Cell culture and induction of maturation of HL-60 cell line

HL-60 cells (ATCC-CCL-240, obtained in Nov. 2010) [32] were differentiated to granulocytes by retinoid acid (RA, Sigma-Aldrich Chemical Company, St. Louis, MO, USA) at a concentration of 10^–7^ M for 5 days [33]. Both PMNs and HL-60 (RA) cells were subcultured in RPMI-1640 medium (Gibco-BRL, Grand Island, New York, USA) supplemented with 10% fetal bovine serum (Thermo Scientific, Logan, Utah, USA).

### Expression, purification, and identification of human full-length recombinant SSB/La protein

Expression and purification of histidine-tagged rSSB protein from *E*. *coli* was performed as described in our previous report [30]. Immobilized polymyxin B gel (Thermo Scientific, Rockford, IL, USA) was used to remove any potentially contaminating lipopolysaccharide (LPS) in the preparations, with an efficiency of greater than 99.95%. The concentrations of endotoxin and protein in the purified rSSB preparations were determined using a Limulus amebocyte lysate assay (Lonza, Walkersville, MD, USA) and a bicinchoninic acid assay (Thermo Scientific, Rockford, IL, USA), respectively. The rSSB molecule was separated in 12% SDS-PAGE, followed by reactions with Coomassie blue, purified human polyclonal anti-SSB antibodies, mouse monoclonal antibodies against histidine tag (anti-HisTag), or mouse monoclonal anti-SSB antibodies.

### Purification of human polyclonal anti-SSB autoantibodies

Human polyclonal anti-SSB antibodies were purified as described in our previous study [30]. The preparations were stored at -80°C until use.

### Cell stimulation

Cells were treated with rSSB at 37°C at the indicated doses and times. LPS (100 ng/ml, Sigma-Aldrich) or TNF-α (20 ng/ml, ProSpec, NJ, USA) was used as the PMN stimulant for the positive controls, and bovine serum albumin (BSA, Sigma-Aldrich) as a negative protein control. In some experiments, PMNs were preincubated with polymyxin B (100 μg/ml, Sigma-Aldrich) to inactivate LPS before stimulation with rSSB or LPS. In other experiments, rSSB was pretreated with proteinase-K (Sigma-Aldrich), RNase A (Sigma-Aldrich) or boiling and freezing for 1 hour before incubation with the cells. In TNF-α priming experiments, PMNs were pretreated with/without TNF-α (20 ng/ml) for 30 minutes and then stimulated with/without rSSB (10 μg/ml) for 2 hours. In immune complex (IC) experiments, the cells were treated with preformed ICs made up of rSSB and purified anti-SSB antibodies for 2 hours. In endosome inhibition experiments, the cells were pretreated with different concentrations (5–50 μg/ml) of chloroquine (CQ, Sigma-Aldrich) or 30 mM NH_4_Cl (Sigma-Aldrich) for 1 hour, followed by stimulation with rSSB at the indicated times.

### IL-8 quantitation in cultured supernatants by ELISA

Human IL-8 protein levels in different cultured supernatants were measured using a commercially available ELISA kit obtained from R&D Systems (Minneapolis, MN, USA) according to the manufacturer’s instructions.

### Q-PCR for IL-8 mRNA expression

Total cell RNAs were extracted using the RNeasy mini kit (Qiagen, Hilden, Germany) and then reversely transcribed into cDNA. Q-PCR was conducted using a StepOne Real-Time PCR System (Applied Biosystems, Foster, CA, USA). The primer pair for human IL-8 (forward 5'-atg act tcc aag ctg gcc gtg gct-3’; reverse 5'-tct cag ccc tct tca aaa act tc-3’) were purchased from BioBasic (Markham, Canada). The relative abundance of IL-8 mRNA was calculated using the ΔΔCt method and normalized to cyclophilin.

### Detection of FITC-rSSB uptake by flow cytometry and fluorescence microscopy

Fluorescein isothiocyanate (FITC) was purchased from Sigma-Aldrich and conjugated to rSSB or BSA according to the manufacturer’s protocols. Subsequently, the cells (1x10^6^ /ml) were incubated with 10 μg/ml of FITC-rSSB or FITC-BSA at 4°C or 37°C for the indicated times. After washing, 0.4% trypan blue was added to quench the extracellular FITC fluorescence. The percentage of positive cells was analyzed using a BD FACSCalibur Flow Cytometer (San Jose, CA, USA). We used propidium iodide to stain the dead cells. To study the effect of different molecules on the uptake of FITC-rSSB, HL-60 (RA) cells were incubated with medium alone or with FITC-rSSB (40 μg/ml) in the presence of medium, LPS, non-specific IgG or purified human polyclonal anti-SSB antibodies for 15 minutes, followed by flow cytometric analysis. The FITC-rSSB uptake by HL-60 cell lines was also observed under fluorescence microscopy.

### Detection of signaling pathways in PMNs and HL-60 (RA) cells

For detection of MAPK activation, whole cell lysate was subjected to 12% SDS-PAGE and transferred to a PVDF membrane. Rabbit polyclonal antibodies as the primary antibodies against signaling molecules including ERK (p42/p44), phospho-p42/p44, p38, phospho-p38, JNK, and phospho-JNK, were purchased from Cell Signaling Technology (Danvers, MA). Mouse polyclonal antibodies against GAPDH were purchased from Sigma-Aldrich. After incubation with the appropriate primary and horseradish peroxidase-conjugated secondary antibodies, blots were developed using SuperSignal West Pico reagents (Thermo Scientific, Logan, Utah, USA).

To identify the critical MAPK signaling pathway activated by rSSB, cells were preincubated with SB203580 (specific p38 inhibitor, Sigma-Aldrich), PD98059 (specific MEK-1 inhibitor, Sigma-Aldrich), or pertusis toxin (specific Gαi-protein-coupled receptor inhibitor, Calbiochem, San Diego, CA, USA) for 1 hour, and then incubated with rSSB or LPS for 2 hours.

### NF-κB binding activity in nuclear extracts

Nuclear extracts were isolated using Thermo Scientific NE-PER Nuclear and Cytoplasmic Extraction Kits. The binding activity of NF-κB subunits p65 and p50 in the nuclear extract were detected using TransAM NF-κB Kits (Tokyo, Japan) according to the manufacturer’s instructions.

### Statistical analysis

Results are presented as the mean ± S.D. The Student’s t-test was used for comparisons of continuous variables between two groups. One-way analysis of variance (ANOVA) with Bonferroni correction for multiple comparisons was applied for more than two groups. A p-value of 0.05 or lower was considered to indicate statistical significance.

## Results

### Identification of recombinant human SSB

**Fig 1:**
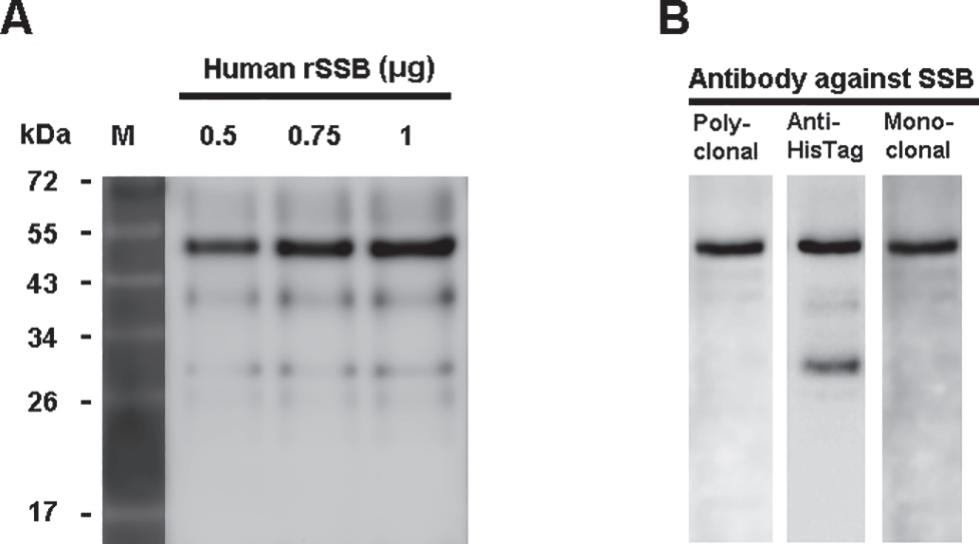
Identification of recombinant human SSB protein. Recombinant human Sjögren's syndrome antigen B (rSSB) preparations were analyzed in 12% SDS-PAGE followed by Coomassie blue staining (**A**), and Western blotting reaction (**B**) with purified human polyclonal anti-SSB antibodies, mouse monoclonal anti-histidine tag antibodies (anti-HisTag) or mouse monoclonal antibodies against human SSB. A distinct band with a molecular weight around 50 kDa was identified as recombinant human SSB protein.

To identify the prepared rSSB, the preparations (0.5 to 1 μg) were subjected to 12% SDS-PAGE. As shown in Fig 1A, a prominent band with a molecular weight around 50 kDa was found in Coomassie blue staining. The higher molecular weight than human SSB (48 kDa) was due to the inserted polyhistidine tag in the molecule. The purified molecule was proven to be histidine-tagged recombinant human SSB by Western blotting using mouse monoclonal anti-HisTag antibodies, human polyclonal anti-SSB antibodies, and mouse monoclonal anti-SSB antibodies as probes (Fig 1B).

### Recombinant SSB stimulated IL-8 production from normal human PMNs

**Fig 2:**
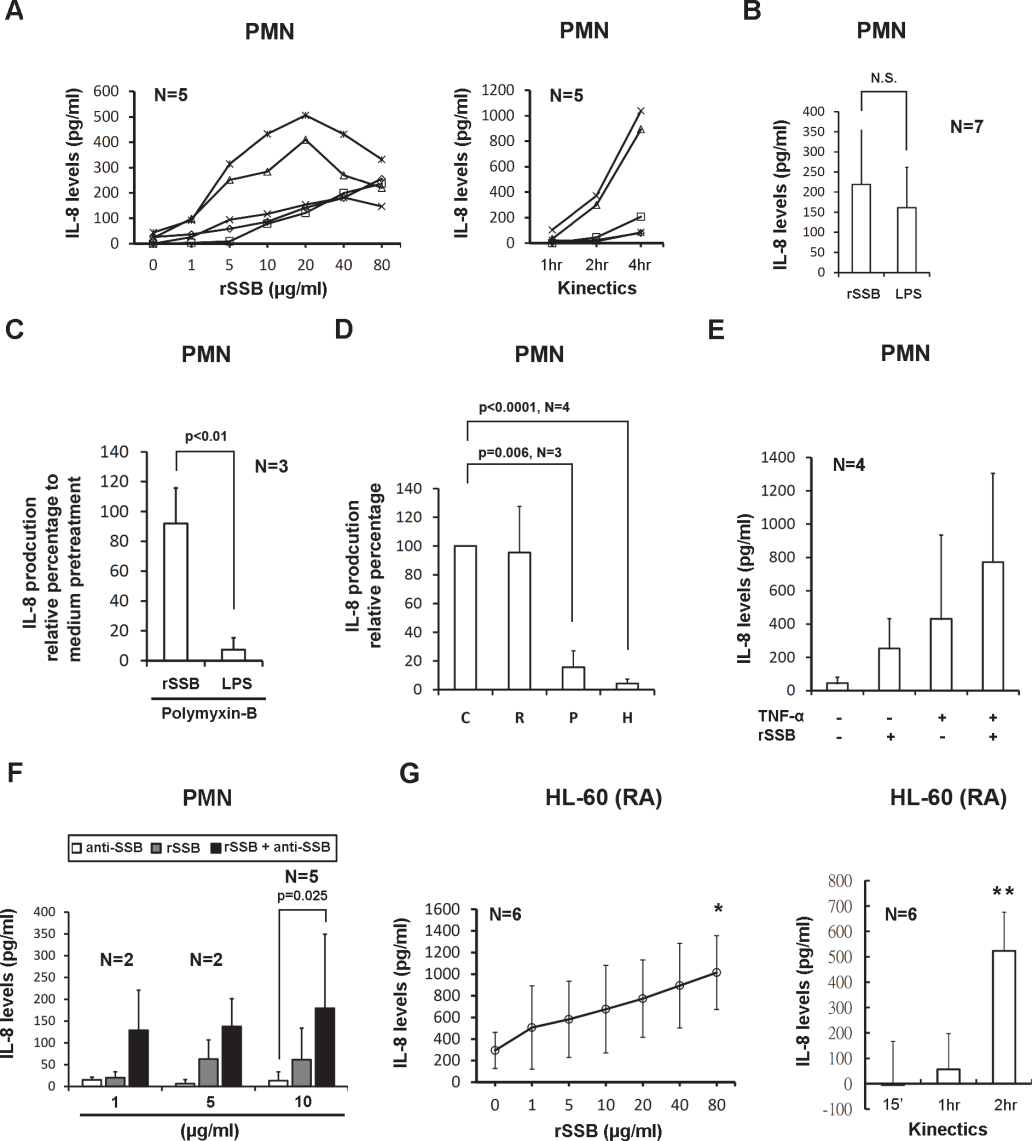
IL-8 production from normal human PMNs and retinoid acid-induced granulocytic differentiated HL-60 (RA) cells. Cells (1x10^6^ cells/ml) were treated with different stimuli and IL-8 levels in culture supernatants were measured. **A,** Dose-responsive (left panel) and time-dependent (right panel) effects of rSSB on IL-8 production from normal human PMNs. Incubation with rSSB (10 μg/ml) for 2 hours were chosen in the ongoing experiments. **B,** Comparison of IL-8 production from normal PMNs after incubation with rSSB (10 μg/ml) and LPS (100 ng/ml) for 2 hours. Medium effect was subtracted in both groups. **C,** The effect of polymyxin-B on rSSB- or LPS-stimulated IL-8 production from normal PMNs. **D,** The effect of different modifications of rSSB molecules on IL-8 production from normal PMNs, including: C: controls (intact rSSB molecule as 100%), R: RNase A-digested rSSB (n = 2), P: proteinase-K digested rSSB (n = 3) and H: heat-denatured rSSB (n = 4). **E,** An additive effect of TNF-α pretreatment on rSSB-induced IL-8 production from normal PMNs. **F,** Effect of rSSB, anti-SSB antibody and rSSB-anti-SSB immune complex on IL-8 production from normal PMNs. **G,** The dose-responsive (left channel) and time-dependent (right channel) effects of rSSB (10 μg/ml) on IL-8 production from HL-60 (RA) cells. *p<0.05; **p<0.001

Incubation of normal human PMNs (1x10^6^ cells/ml) with rSSB resulted in a dose- (1–20 μg/mL) and time- (1–4 hours) dependent increase in IL-8 production (Fig 2A). We found that 10 μg/ml of rSSB (219.4±152.5 pg/ml) was as effective as 100 ng/ml of LPS (160.8±100.6 pg/ml) in stimulating IL-8 production from normal PMNs (Fig 2B). In contrast, BSA as a protein control failed to enhance IL-8 production in 3 normal control, even at the dose of 80 μg/ml (54.14 ±75.35 pg/ml in medium group vs. 47.56±66.62 pg/ml in BSA group). This IL-8 stimulating activity of rSSB was not due to endotoxin contamination during preparation since polymyxin B (100 μg/ml) had no effect on rSSB-induced IL-8 production from PMNs (Fig 2C). In addition, by Limulus amebocyte lysate (*LAL*) detection method, the average endotoxin level in the rSSB preparations were 0.0032 EU/10g/ml.

### Characteristics of recombinant SSB responsible for enhancing IL-8 production from PMNs

To further characterize the IL-8 stimulating activity of rSSB, we performed enzyme-digestion or heat-denaturation of rSSB. The results showed that an intact protein structure of rSSB mediated the activity as proteinase K treatment and heat-denaturation, but not RNase A treatment of rSSB reduced the rSSB activity to produce IL-8 (Fig 2D). In brief, the rSSB protein molecule *per se*, but not contaminating LPS or RNA, was responsible for the IL-8 stimulating activity.

### TNF-α pretreatment augmented IL-8 production from rSSB-stimulated PMNs

Since TNF-α is a potent proinflammatory cytokine, we next investigated whether TNF-α would affect rSSB-induced IL-8 production from PMNs. As shown in Fig 2E, rSSB (10 μg/ml) or TNF-α (20 ng/ml) alone enhanced IL-8 production (254.2 ±178.2 pg/ml vs. 431.2±503.2 pg/ml). TNF-α pretreatment further augmented rSSB-induced IL-8 production (771.7±532.7 pg/ml) from normal human PMNs. These results suggest that rSSB and TNF-α augment IL-8 production from PMNs via different pathways.

### rSSB induced IL-8 production from HL-60 (RA) cells

Similar to PMNs, rSSB induced IL-8 production from HL-60 (RA) cells in a dose- and time-dependent manner (Fig 2G).

### Uptake of rSSB by PMN

**Fig 3:**
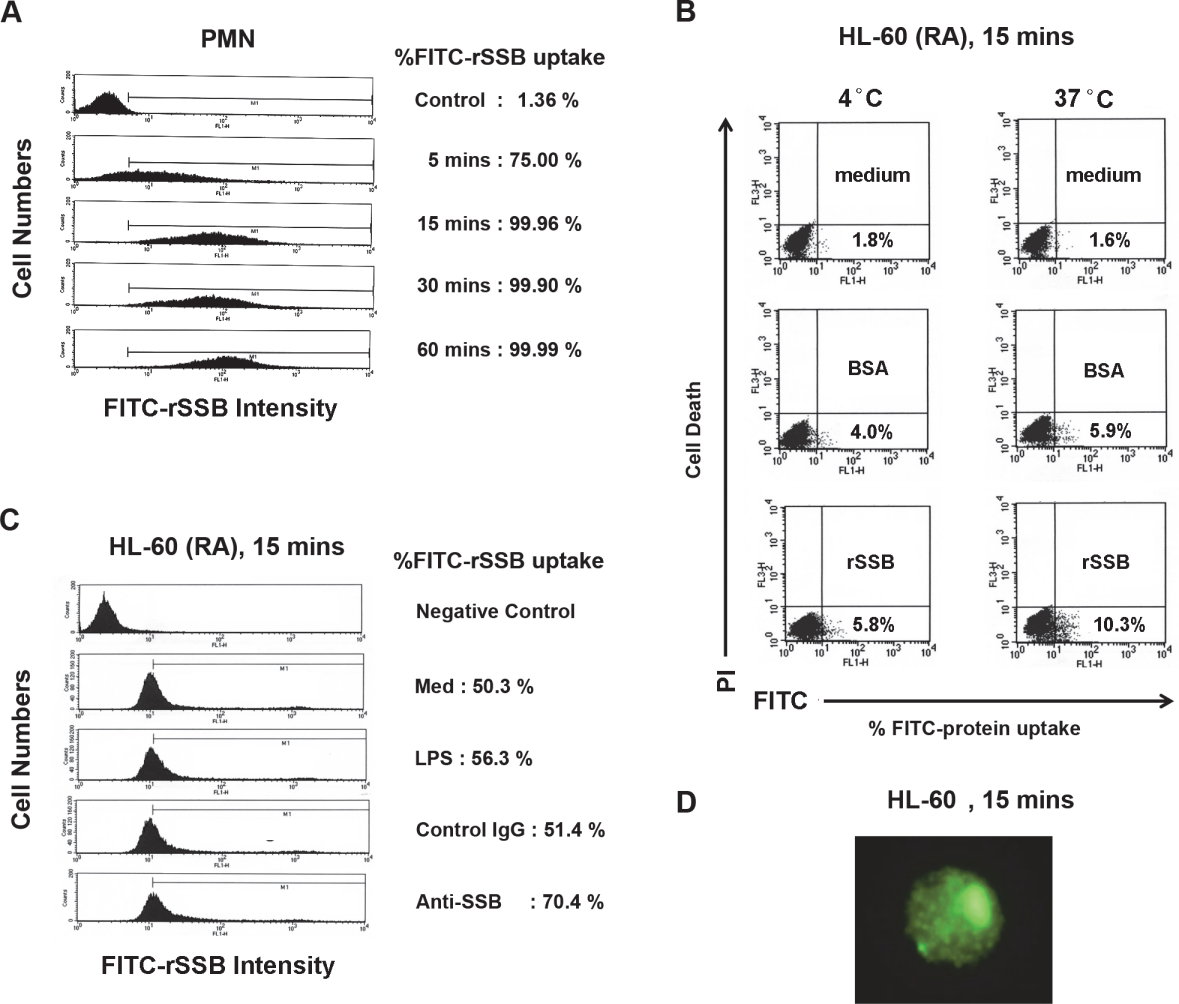
Uptake of FITC-rSSB by normal human PMNs and HL-60 cells. **A,** Kinetic uptake of FITC-rSSB (10 μg/ml) by normal human PMNs after incubation for 5–60 minutes. The percentage of FITC-rSSB uptake was analyzed by flow cytometry after quenching the extracellular FITC fluorescence by adding 0.4% trypan blue. **B,** Comparisons of FITC-BSA (10 μg/ml) and FITC-rSSB (10 μg/ml) uptake by HL-60 (RA) cells after incubation for 15 minutes at 4°C or 37°C. The percentage of cell death was also compared at 4°C and 37°C for 15 minutes by propidium iodide (PI) staining. **C,** Comparisons of the percentage of FITC-rSSB (40 μg/ml) uptake by HL-60 (RA) cells in the presence of medium (Med), LPS (100 ng/ml), non-specific IgG (10 μg/ml) or purified human polyclonal anti-SSB antibody (10 μg/ml) for 15 minutes. **D,** Uptake of FITC-rSSB by HL-60 cells was observed with fluorescence microscopy. A representative case of 2–3 independent experiments is shown in **A-D**.

To investigate whether SSB autoantigens enter into the cell interior or merely bind to the cell surface of PMNs to activate IL-8 production, normal PMNs were incubated with FITC-rSSB for different time periods. Uptake, but not mere attachment, of FITC-rSSB by PMN began at 5 minutes and reached a maximal level after 15 minutes of incubation (Fig 3A). As shown in Fig 3B, the uptake of rSSB was specific and irrelevant to cell death, and appeared higher at 37° than that at 4°. However, the difference in our small sample size did not reach a statistically significant level (p = 0.21). More samples are needed to determine whether uptake of rSSB is energy-dependent. Likely, although it seemed that human polyclonal anti-SSB autoantibodies enhanced FITC-rSSB uptake at 15 minutes compared to medium, LPS or IgG (Fig 3C), more experiments are needed to draw any conclusion (p = 0.55, compared with medium control). The uptake of FITC-rSSB by HL-60 cells was also observed by fluorescence microscopy (Fig 3D).

### Enhanced PMN IL-8 production by SSB-anti-SSB immune complex

As anti-SSB antibody seemed enhanced FITC-rSSB uptake by HL-60 (RA) cells (Fig 3C), we next investigated whether anti-SSB autoantibody or preformed SSB-anti-SSB ICs influenced IL-8 production from human PMNs and HL-60 (RA) cells. As shown in Fig 2F, anti-SSB antibody (10 μg/ml) exerted a negligible effect on IL-8 production from normal PMNs. However, rSSB-anti-SSB ICs markedly enhanced IL-8 production. The synergic effect of SSB-anti-SSB ICs on IL-8 production was also observed in HL-60 (RA) cells (S1 Fig). We speculate that anti-SSB antibody at this concentration might merely facilitate the uptake of SSB molecules by these cells (Fig 3C), but have no effect on IL-8 production (Fig 2F). Instead, uptake of SSB-anti-SSB ICs by phagocytes through Fc-gamma receptors (FcγRs) exerts a synergistic effect on IL-8 production from these cells (Fig 2F).

### Chloroquine and NH_4_Cl decreased SSB-induced IL-8 gene expression in HL-60 (RA) cells and human PMNs

**Fig 4:**
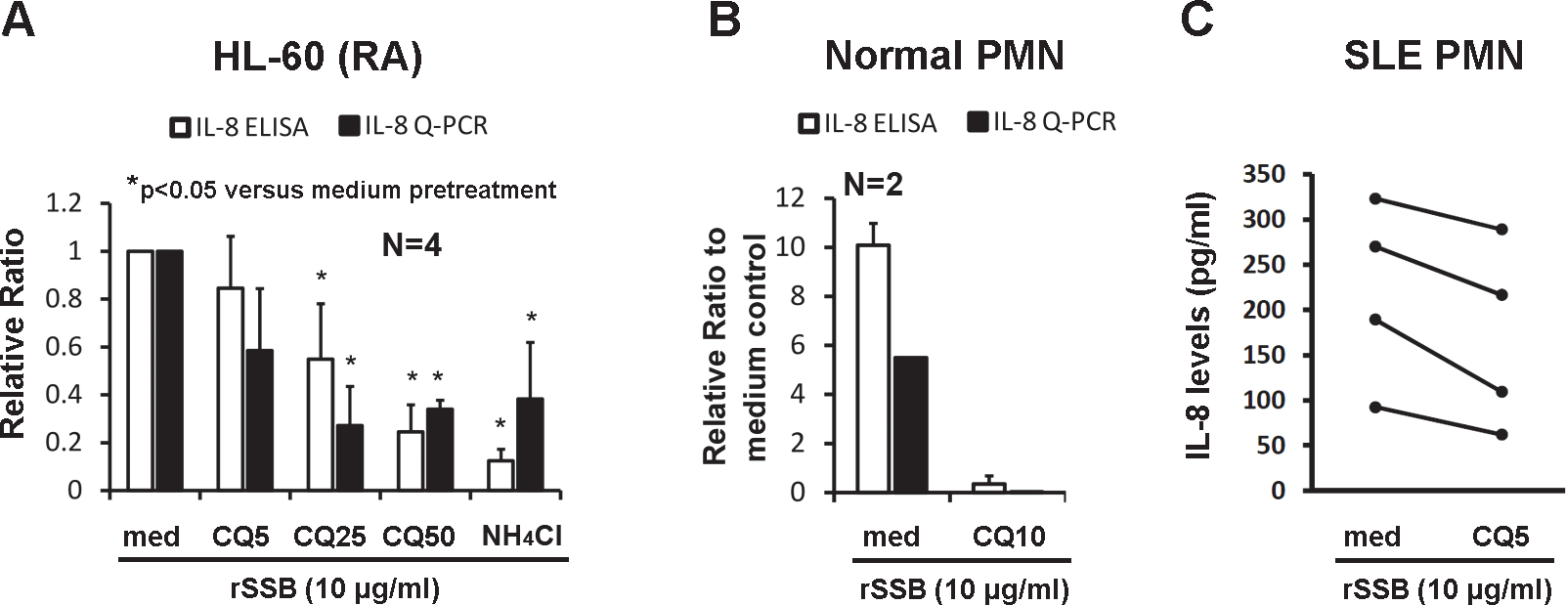
Effect of chloroquine (CQ) and NH_4_Cl on IL-8 gene expression in HL-60 (RA) cells and human PMNs. **A,** The effect of CQ (5–50 μg/ml) and NH_**4**_Cl (30 mM) pre-incubation on rSSB-induced IL-8 gene expression in HL-60 (RA) cells was determined by ELISA (after 2 hours of stimulation) and Q-PCR (after 1 hour of stimulation). Relative ratios were calculated compared with the medium pretreatment group (set at 1.0). **B,** Effect of CQ (10 μg/ml) pre-incubation on rSSB-stimulated IL-8 mRNA expression (after 1 hour of stimulation) and IL-8 protein secretion (after 2 hours of stimulation) by normal PMNs. Relative ratios were calculated compared to medium control. **C,** Effect of CQ (5 μg/ml) pre-incubation on IL-8 production from rSSB-incubated PMNs from patients with SLE.

As endosomes contain internalized molecules, we compared the effect of endosome acidification inhibitors on rSSB-induced IL-8 gene expression in HL-60 (RA) cells (Fig 4A). Pretreatment with different concentrations of CQ (5–50 μg/ml) and NH_4_Cl (30 mM) displayed a dose-responsive inhibitory effect on rSSB-induced IL-8 gene expression. Likewise, the inhibitory effect of CQ on IL-8 gene expression was observed in normal human PMNs (Fig 4B). Since hydroxychloroquine (HCQ) is frequently used in patients with SLE, we evaluated the effect of CQ on rSSB-induced IL-8 production from the PMNs of lupus patients. Pretreatment of lupus PMNs with 5 μg/ml of CQ reduced the rSSB-induced IL-8 production by 22.7% compared to the medium pretreatment group (Fig 4C).

### Signaling induced by rSSB in PMNs was through MAPK pathways and NF-κB activation

**Fig 5:**
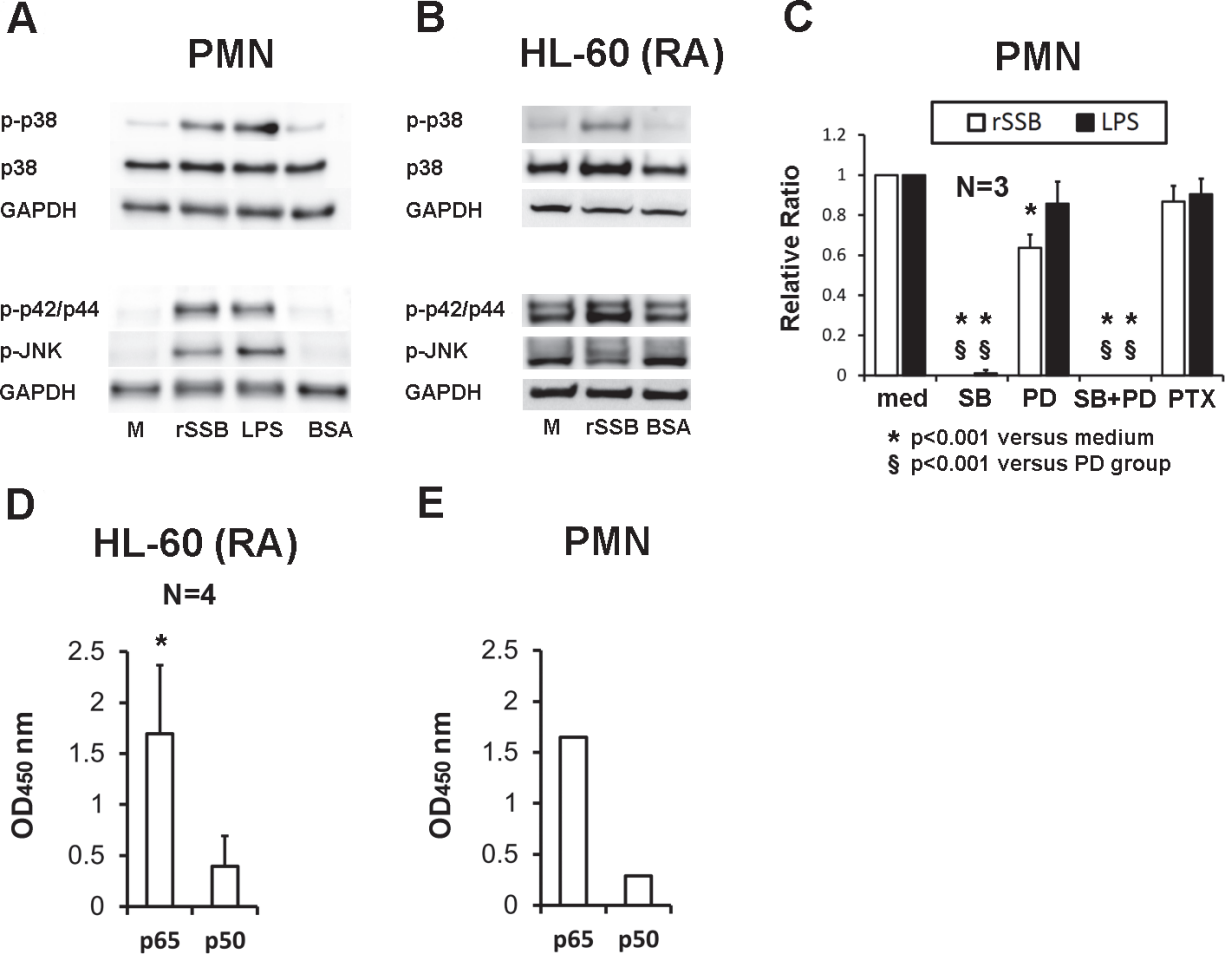
Signaling pathways for rSSB-induced IL-8 production in normal human PMNs and HL-60 (RA) cells. **A,** Activation and phosphorylation of p38, ERK1/2 and JNK MAPK pathways by rSSB (10 μg/ml) in normal human PMNs. **B**, Activation and phosphorylation of p38 and ERK1/2 MAPK pathways by rSSB (10 μg/ml) in HL-60 (RA) cells. A representative blot of 3 independent experiments is shown in (**A**) and (**B**). **C,** The effects of specific inhibitors for p38 (SB203580, SB 10 μM), MEK-1 (PD98059, PD, 10 μM), p38 and MEK-1 (SB + PD, 10 μM each), and Gαi-protein-coupled receptors (pertussis toxin, PTX, 100 ng/ml) on rSSB (10 μg/ml)- or LPS (100 ng/ml) induced IL-8 production were compared. The relative ratio of IL-8 production in the medium pretreatment group was set at 1, and multiple comparisons were adjusted by Bonferroni correction. **D,** Nuclear translocation of NF-κB subunits p65 and p50 induced by rSSB (10 μg/ml) at 1 hour compared to medium control in HL-60 (RA) cells. *p<0.05. **E,** Nuclear translocation of NF-κB subunits p65 and p50 induced by rSSB (10 μg/ml) at 1 hour compared to medium control in normal human PMNs. A representative result of 2 independent experiments is shown in **E**.

To explore the signaling pathway transduced by rSSB, immunoblotting of total cell lysates of rSSB-activated normal human PMNs (Fig 5A) and HL-60 (RA) cells (Fig 5B) were performed. MAPK pathways including p38 and ERK1/2 in both cells and JNK on PMNs were activated by rSSB. Furthermore, by pre-incubating normal PMNs with different specific inhibitors (Fig 5C), we found that the p38 specific antagonist SB203580 completely inhibited the IL-8 enhanced activity of rSSB (0±0%, *p*<0.001) and LPS (1.0%±1.8%, *p*<0.001) compared to the medium controls (relative ratio was set at 1). In contrast, the MEK-1 specific antagonist PD98059 exerted a negligible effect on LPS-induced IL-8 production from normal PMNs (85.7%±11.1%, *p* = 0.168), but it reduced the rSSB-induced IL-8 production (63.7%±6.8%, *p*<0.001). The Gαi-protein-coupled receptor inhibitor pertussis toxin had no effect on rSSB- or LPS-induced IL-8 production from normal PMNs. Moreover, rSSB activated the nuclear translocation of NF-κB subunit p65 and p50 at 60 minutes in both HL-60 (RA) cells (Fig 5D) and normal human PMNs (Fig 5E).

## Discussion

There are several original findings in this study. First, rSSB enhances IL-8 gene expression in normal human PMNs in a time- and dose-dependent manner. Second, rSSB transduces p38, ERK 1/2 and JNK MAPK signaling and NF-κB subunit p65 and p50 nuclear translocation that is responsible for rSSB-induced IL-8 gene expression in human PMNs. Third, TNF-α exerts an additive effect whereas the SSB-anti-SSB IC exerts a synergistic effect on rSSB-induced IL-8 production. Finally, CQ reduced the rSSB-induced IL-8 production from neutrophils in both normal controls and patients with systemic lupus erythematous.

Cell death and delayed clearance of intracellular antigens are involved in the development of autoimmunity [16,17]. Many endogenous adjuvant molecules and nuclear autoantigens are released from excessive tissue damage and cell death [19–21]. As TNF-α and IL-8 play a critical role in amplifying the inflammatory response and sustaining tissue damage and cell death, our results suggest that released SSB from dead cells might act as a danger molecule to induce IL-8 production from PMNs.

Gallucci et al. [18], Shi et al. [22], and Matzinger et al. [34] reported that danger signals from tissue damage and cell death can alert the immune system to responses. Currently, intracellular molecules such as ATP [21], UTP [20], uric acid [22], and heat-shock proteins [35,36] are recognized to be endogenous danger signals that activate immune cells by themselves or in conjunction with TNF-α. Moreover, some autoantigens have been reported to mediate pathologic immune responses in systemic autoimmune diseases [23–29]. For example, the carboxy-terminal domain of tyrosyl-tRNA synthetase (TyrRS, 1 nM) has been shown to exhibit chemotactic activity for monocytes and PMNs and to stimulate the production of myeloperoxidase, TNF-α, and tissue factor. The amino-terminal domain of TyrRS (1 nM) has been reported to induce PMN migration [27], and human myelin basic protein (10–30 μg/ml) has been reported to induce a dose-dependent release of interferon-γ, TNF-α and IL-10 from the mononuclear cells of patients with multiple sclerosis [28]. In addition, HSP60 (10 μg/ml) has been reported to induce significant amounts of TNF-α, IL-1 and IL-10 from peripheral blood mononuclear cells of patients with juvenile dermatomyositis [29]. As rSSB (10 μg/ml), alone or in combination of TNF-α, induces IL-8 production from normal neutrophils, it is suggested that SSB might be a new endogenous danger molecule that can activate PMNs to produce IL-8. Moreover, SSB-anti-SSB ICs induce a much higher level of IL-8 production from PMNs.

In our study, the concentration of rSSB (10 μg/ml) we used to stimulate cells was similar to that of other autoantigens mediating immune responses [28, 29]. However, data on the concentration of SSB in vivo was not addressed in the literature we reviewed. As SSB has been very abundant in the cell (20 million copies per cell) [3], the estimated intracellular concentration of SSB is around 400 μg/ml assuming that average cell volume to be 4 billionths of a cubic centimeter [37]. Moreover, SSB expression may be upregulated in inflamed tissues [38]. Hence, the possible local concentration of released SSB after cell death was estimated to be high enough (in the order of μg/ml) to induce biologic effects. It is also postulated that the high expression of SSB in labial salivary gland ductal cell might contributed to the local immune response in pSS. [38].

Neutrophils have been suggested to play a central role in initiation and perpetuation of aberrant immune responses in systemic autoimmune diseases, including SLE and rheumatoid arthritis [39]. Although the role of neutrophils in the pathogenesis of pSS remained elusive, there are several findings that support neutrophils play a part in the pathogenesis of pSS. First, we demonstrated human SSB as well as SSB-anti-SSB IC induced IL-8 production from normal neutrophils. Second, neutropenia is frequently noted in pSS (33%) and is closely associated with anti-SSB antibody [40]. As SSB can be expressed in the surface membrane of human neutrophils and purified anti-SSB antibody increased apoptosis and IL-8 production of these cells [30], the abnormal SSB antigen expression in neutrophil membranes may induce the synthesis of autoantibodies against these cells and cause their lysis [40]. This also supports a possible role of neutrophil in the pathogenesis of pSS. Finally, neutrophils are activated in pSS [41].

**Fig 6:**
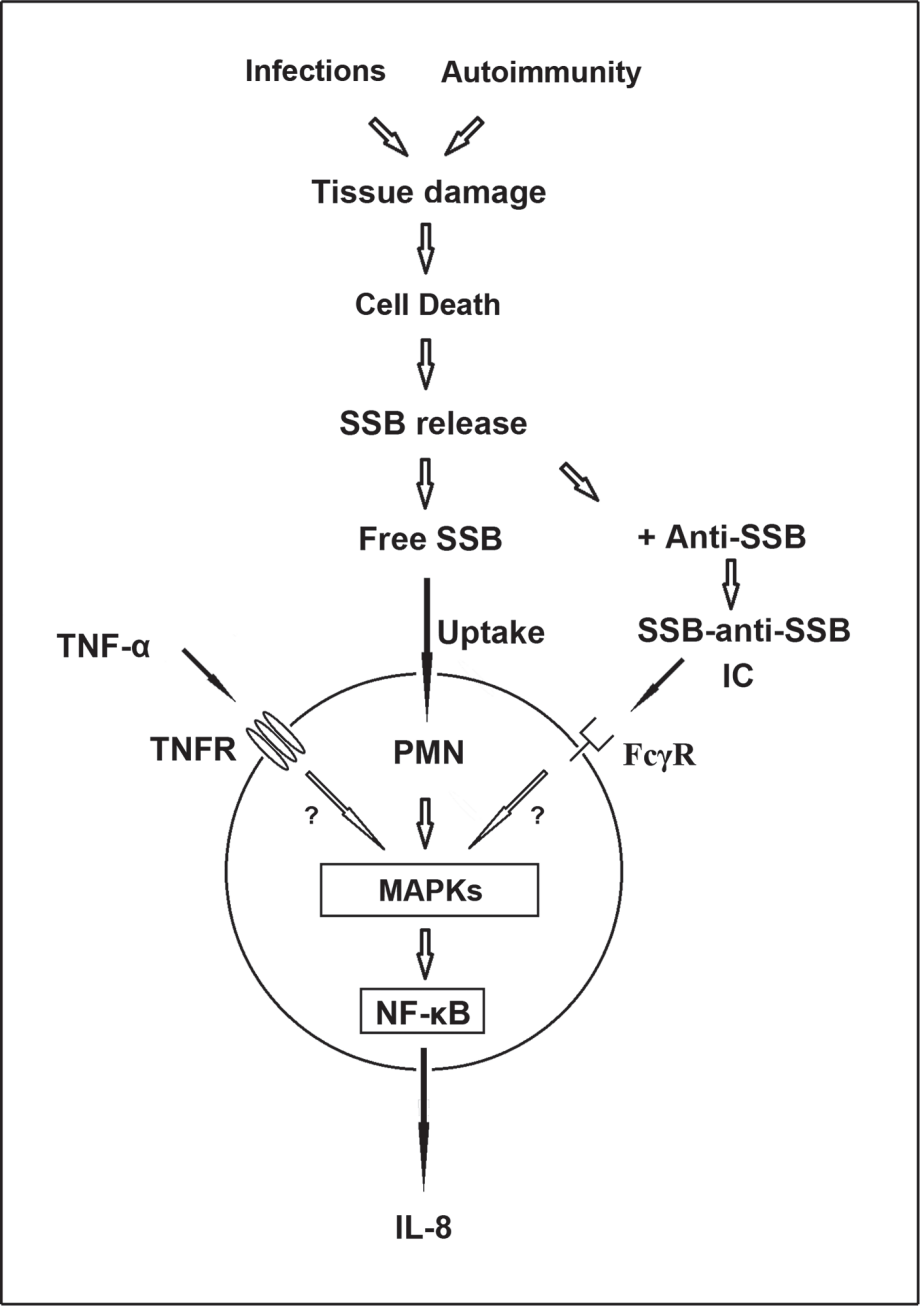
Schematic diagram illustrating the interactions of SSB with PMNs and the signaling pathways. During infections or autoimmune responses, SSB molecules are released from damaged tissues and dead cells. The free SSB molecules activate PMN via MAPK pathways and NF-κB nuclear translocation to transcribe the IL-8 gene. TNF-α and SSB-anti-SSB immune complexes, which may utilize TNF-α receptors (TNFR) and Fc-gamma receptors (FcγR), respectively, augment IL-8 production probably through a final common pathway.

A schematic diagram illustrating the interaction of SSB with human PMN is shown in Fig 6. After uptake of free rSSB, MAPK signaling (p38, ERK1/2, and JNK) and NF-κB subunit p65 and p50 nuclear translocation were activated to transcribe IL-8 gene expression in human PMNs. In addition, TNF-α and SSB-anti-SSB ICs may utilize TNF-α receptors and FcγRs, respectively, to augment the production of IL-8. These findings are compatible with the well-known intracellular mechanisms for IL-8 production [42–44]. As many autoantigens and endogenous adjuvants activate Toll-like receptors [45], nucleotide-binding oligomerization-like receptors [46] and chemokine receptors [24], it would be interesting to investigate the role of these receptors in the rSSB-induced activation of PMNs. The basic mechanisms by which rSSB stimulates IL-8 production and correlations of serum cytokines levels with concentrations of SSB as well as SSB-anti-SSB IC levels in patients with pSS are now under investigation. Further larger studies are also needed to explore the role of CQ/HCQ in the rSSB-induced activation of PMNs from patients with SLE.

## Conclusions

SSB might act as an endogenous danger molecule to enhance IL-8 gene expression of PMNs. This not only expands the biological function of SSB, but supports the concept that autoantigens *per se* contributes to the generation of autoimmunity in genetically susceptible subjects.

## Supporting Information

S1 Dataset Individual data points in this study. (XLS)

S1 Fig IL-8 production in retinoid acid-induced granulocytic differentiated HL-60 cells after stimulation with rSSB-anti-SSB immune complex. Cells (1x10^6^ cells/ml) were treated with rSSB, anti-SSB antibody and rSSB-anti-SSB immune complex at indicated concentration for 2 hours. IL-8 levels in culture supernatants were measured.
(TIF)
