# Supplementary figures and images for: Sjögren’s Syndrome Antigen B Acts as an Endogenous Danger Molecule to Induce Interleukin-8 Gene Expression in Polymorphonuclear Neutrophils
Source: PLoS One. 2015 Apr 27;10(4):e0125501. doi: 10.1371/journal.pone.0125501 (PMC4411107; doi:10.1371/journal.pone.0125501)

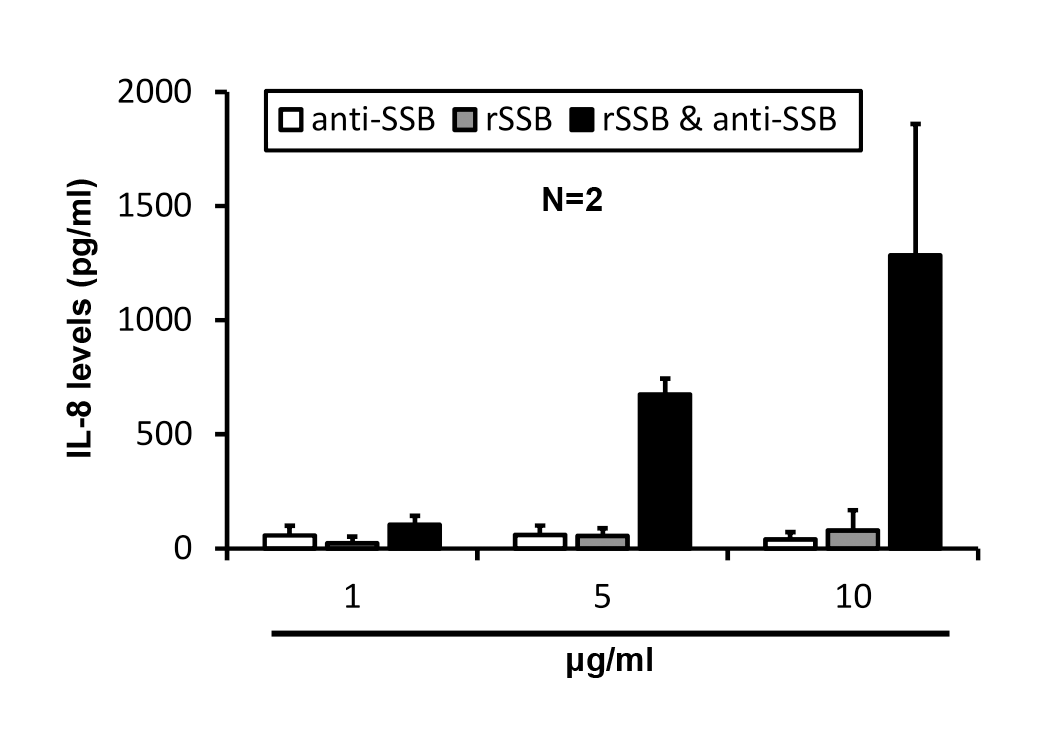

Supplement: S1 Fig — Cells (1x10^6^ cells/ml) were treated with rSSB, anti-SSB antibody and rSSB-anti-SSB immune complex at indicated concentration for 2 hours. IL-8 levels in culture supernatants were measured. (TIF) [file pone.0125501.s002.tif]
